# Multimedia health education to alleviate anxiety in patients with vitreous floaters

**DOI:** 10.3389/fpubh.2026.1769080

**Published:** 2026-06-09

**Authors:** Qing Feng, Yalin Jiang, Yuan Li, Xincheng Sun

**Affiliations:** Department of Ophthalmology, The Second People's Hospital of Changzhou, The Third Affiliated Hospital of Nanjing Medical University, Changzhou, Jiangsu Province, China

**Keywords:** anxiety, health education, multimedia, visual-related quality of life, vitreous floaters

## Abstract

**Background:**

Vitreous floaters (VF) are common and typically benign but may cause significant anxiety due to misinterpretation of symptoms. This study aimed to evaluate the effectiveness of structured multimedia health education in reducing anxiety among patients with VF and to identify factors associated with anxiety severity.

**Methods:**

This prospective quasi-randomized controlled study was conducted between December 2023 and April 2025. Patients with symptomatic VF were allocated to an enhanced multimedia education group or a standard care group based on calendar week. Anxiety was assessed using the State-Trait Anxiety Inventory (STAI-state), and visual-related quality of life (VRQoL) was measured using the National Eye Institute Visual Function Questionnaire-25 (NEI VFQ-25) at baseline and 3 months. Between-group differences in STAI-state change (ΔSTAI-state) were analyzed, and analysis of covariance (ANCOVA) was performed adjusting for baseline values. Multivariable logistic regression was used to identify factors associated with moderate-to-severe anxiety.

**Results:**

A total of 309 participants completed the study. Both groups showed significant reductions in STAI-state scores; however, the reduction was significantly greater in the multimedia group (ΔSTAI-state: −5.22 vs. −1.09), with a between-group difference of −4.13 (95% CI: −5.21 to −3.05, *p* < 0.001). ANCOVA confirmed significantly lower follow-up anxiety levels in the intervention group (*p* < 0.001). No significant between-group differences were observed in VRQoL. Patients with higher education levels exhibited greater anxiety reduction. Factors such as older age, higher education, higher trait anxiety, and diabetes were independently associated with moderate-to-severe anxiety.

**Conclusions:**

Structured multimedia health education is more effective than standard counseling in reducing anxiety among patients with VF. These findings highlight the importance of optimized physician–patient communication as a non-invasive strategy to address psychological distress in benign ophthalmic conditions.

## 1 Introduction

Symptomatic vitreous floaters (VF) are a common ophthalmic complaint, typically resulting from the introduction of exogenous substances into the vitreous body due to hemorrhage or inflammation, or from an entoptic phenomenon caused by the molecular rearrangement of hyaluronic acid and collagen macromolecules (1). Entoptic images caused by vitreous opacities cast shadows on the retina, resulting in the visual perception of typically gray, but also black or translucent, linear, circular, or nodular patterns. These patterns drift synchronously with eye and head movements owing to the dynamic displacement of vitreous opacities within the gel-like medium of the eye (2). Floaters may be present in up to 77% of the population, as evidenced by population-based epidemiological studies (3, 4). In recent years, Nd:YAG laser vitreolysis has garnered significant clinical interest for the treatment of VF. Although this technique offers multiple potential advantages (2), the currently available evidence guiding clinical decision-making remains limited and inconclusive. While pars plana vitrectomy (PPV) is an effective method for treating vitreous opacities, this procedure exposes the retina to substantial complication risks, including iatrogenic retinal tears and accelerated postoperative cataract formation (5, 6). To date, observation remains the primary management approach for VF, despite advancements in minimally invasive therapies (1).

Several studies have demonstrated the significant psychological impact of VF, with anxiety being a predominant concern (7, 8). A primary source of this anxiety is the patient's perception of their symptoms as indicative of underlying severe ocular pathology, even sight-threatening conditions (9). However, the distress associated with VF extends beyond this fear. Patients frequently report anxiety stemming from the persistent visual intrusion of floaters, which can impair daily activities such as reading and driving, and thereby reduce overall quality of life (10). Furthermore, health-related concerns about the condition's unpredictability and its long-term implications on vision are also significant contributors to psychological burden (11). When patients seek ophthalmic consultation, clinicians, focused on the typically benign prognosis and natural history of the condition, may inadvertently overlook this multifaceted psychosocial distress (12, 13).

Health education, through the provision of evidence-based information and psychosocial support, has been well established as an effective strategy for mitigating both clinical sequelae and psychological burden in patients with certain diseases (12, 14–16). Multimedia health education, which visualizes complex knowledge in an organized and clear manner, is more intelligible than conventional oral education (17). This approach has been shown to enhance patient engagement, improve knowledge retention, and reduce anxiety in several clinical settings. Nevertheless, its application in patients with VF remains largely unexplored. The effectiveness of different communication formats, such as routine verbal counseling vs. structured multimedia education, has not been systematically evaluated in patients with VF. Moreover, the factors associated with anxiety severity in this population have not been fully elucidated. Therefore, the present study aimed to compare the effectiveness of standard post-examination counseling (standard care) with structured multimedia health education (enhanced care) in reducing anxiety among patients with VF. In addition, we sought to identify demographic and clinical factors associated with anxiety severity. By clarifying the role of structured communication in alleviating psychological distress, this study may provide evidence to optimize patient-centered management strategies for individuals with VF.

## 2 Methods

### 2.1 Study design and registration

This was a prospective quasi-randomized controlled study conducted at the Third Affiliated Hospital of Nanjing Medical University between December 2023 and April 2025. The study protocol was prospectively registered at ClinicalTrials.gov (http://clinicaltrials.gov/study/NCT06964867). All procedures were performed in accordance with the Declaration of Helsinki and approved by the institutional ethics committee. Written informed consent was obtained from all participants.

### 2.2 Patients

#### 2.2.1 Inclusion criteria

(1) Patients with VF who visited the Department of Ophthalmology at the Third Affiliated Hospital of Nanjing Medical University, between December 2023 and April 2025; (2) Age between 18 and 80 years; (3) Subjective floaters of endogenous origin confirmed through comprehensive ophthalmic examination, including slit-lamp biomicroscopy, dilated fundoscopy, and optical coherence tomography (OCT); (4) Willingness to participate in the multimedia health education program and complete validated questionnaires, with written informed consent provided.

#### 2.2.2 Exclusion criteria

(1) Secondary VF requiring surgical intervention (e.g., vitreous hemorrhage, retinal breaks, or uveitis); (2) Coexisting ocular pathologies that could confound visual symptoms or vitreoretinal anatomy (e.g., diabetic retinopathy, retinal vascular occlusion, epiretinal membrane, or vitreomacular traction syndrome); (3) History of vitreoretinal surgery (PPV or laser procedures); and (4) Refusal or inability to complete the study protocols.

All examinations were performed by experienced ophthalmologists following standardized clinical protocols. The final diagnosis was determined based on comprehensive evaluation, and patients with pathological findings were excluded to ensure safety.

**Figure 1:**
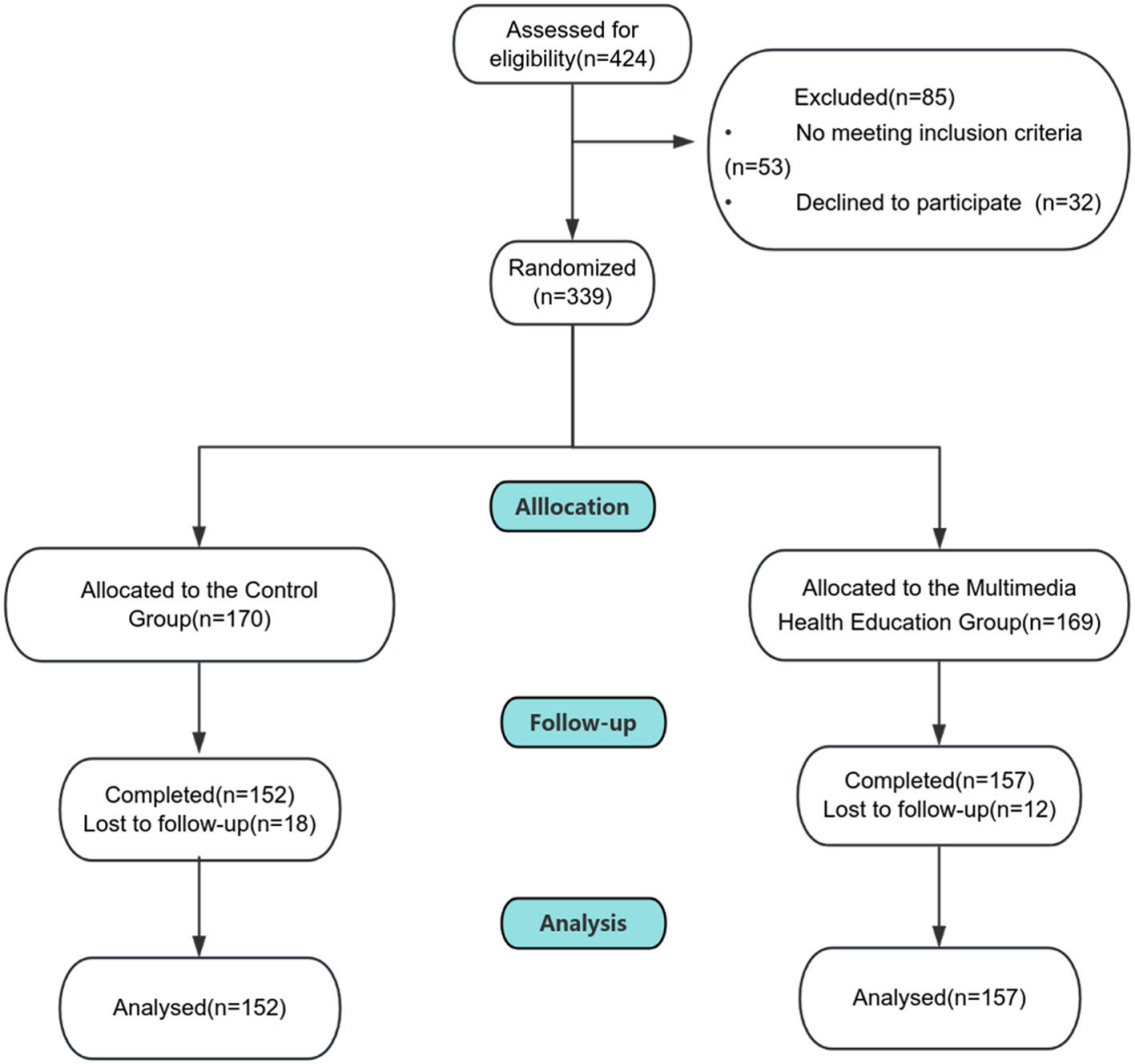
Study flow diagram.

The flow diagram for participant selection is shown in Figure 1.

### 2.3 Allocation and intervention

Participants were allocated using a quasi-randomized temporal approach based on the calendar week of clinic attendance, with patients attending during odd-numbered weeks assigned to the multimedia health education group and those attending during even-numbered weeks assigned to the routine verbal education group. This approach was adopted to facilitate implementation in a real-world clinical setting. Although this method does not represent strict individual-level randomization, consistent eligibility criteria, standardized clinical workflow, and stable clinical personnel were maintained throughout the study period to minimize potential selection bias and time-related confounding.

Multimedia health education was delivered by a single vitreoretinal specialist with over 10 years of clinical experience. This standardized protocol comprised a 30-min didactic session utilizing power point presentations (PPT), systematically covering (1) etiopathogenesis: molecular mechanisms of vitreous syneresis and collagen aggregation; (2) symptomatology: characteristic visual phenomena and differential diagnosis; (3) therapeutic modalities: evidence hierarchy from observation to vitrectomy (including Nd:YAG laser efficacy controversies); and (4) follow-up necessity: red flag symptoms warranting urgent re-evaluation (sudden floaters with photopsia). Multimedia presentations were made using pictures, texts, sounds, and videos. Simple, easy-to-understand language was used to make the study population easy to accept.

The routine verbal education group received routine post-examination counseling delivered by ophthalmologists in the same clinical setting. The counseling typically lasted approximately 5–10 min and included explanation of the benign nature of vitreous floaters, reassurance regarding prognosis, and guidance on symptoms requiring follow-up. The counseling was delivered face-to-face without structured multimedia materials. The personnel and general content were consistent across participants.

### 2.4 Clinical assessment and data collection

All participants underwent comprehensive ophthalmic evaluations, including best-corrected visual acuity (BCVA), slit-lamp biomicroscopy with anterior segment analysis, dilated fundus examination incorporating scanning laser ophthalmoscopy (SLO), and spectral-domain optical coherence tomography (SD-OCT) to exclude vitreoretinal interface pathologies, such as epiretinal membranes and macular holes. The diagnosis of non-pathological vitreous floaters was determined based on these evaluations, and patients with pathological findings were excluded to ensure patient safety.

Demographic and clinical baseline characteristics were systematically recorded, including sex, age, educational attainment, symptom duration, presence or absence of photopsia, time of daily use of electronic devices, comorbidities, shape of floaters, diopter, psychometric anxiety assessment using State-Trait Anxiety Inventory (STAI) and visual-related quality of life (VRQoL) assessments using the National Eye Institute Visual Function Questionnaire-25 (NEI VFQ-25). Education level was categorized into three groups (primary or below, secondary, and college or above), with “college or above” used as the reference category. Baseline anxiety assessment was conducted after completion of the ophthalmic examination and confirmation of diagnosis. The STAI questionnaires were administered by trained personnel using standardized instructions. The same team conducted all assessments to ensure consistency.

### 2.5 Outcomes measures

The primary outcome was the change in STAI score between baseline and 3 months post-intervention. STAI is one of the most commonly used psychological tests to assess anxiety (18), comprising two 20-item subscales state anxiety and trait anxiety: STAI-state evaluating transient emotional responses (“how you feel right now”) and STAI-trait measuring stable anxiety predisposition (“how you generally feel”) (19). This instrument has been extensively applied in populations with chronic diseases, including rheumatoid arthritis, systemic lupus erythematosus, fibromyalgia, and ophthalmic conditions such as chronic glaucoma, diabetic retinopathy, and VF (19). All participants completed the Chinese STAI under standardized conditions (20). The scores for each test ranged from 20 to 80 points, with higher scores indicating higher anxiety (21, 22). Scoring was performed according to the Spielberger's manual criteria. According to the Spielberger manual, the STAI-state and STAI-trait scores are categorized as follows: mild anxiety, 20–39; moderate anxiety, 40–59; and high anxiety, 60–80 (23).

The secondary outcome was the change in NEI VFQ-25 score between baseline and 3 months post-intervention. The Chinese version of the NEI VFQ-25 demonstrates psychometric properties with established reliability and validity, and serves as a validated instrument for assessing VRQoL in ophthalmic populations (24). The scale consists of 12 dimensions (26 items), including one overall health scale and 11 visual-related scales. 26 items were scored from 0 to 100 points. If the patient responded 1, 2, 3, 4, or 5, the scores were assigned 0, 25, 50, 75, or 100 points, respectively. Higher scores indicated better VRQoL, whereas lower scores reflected poorer VRQoL.

The improvement rate of STAI-state was defined as: (baseline score -follow-up score)/baseline score × 100%.

### 2.6 Follow-up

At 3 months, participants completed the STAI-state and NEI VFQ-25 questionnaires under the guidance of trained personnel. The same team administered all assessments using standardized instructions and remained blinded to group allocation.

### 2.7 Statistical analysis

Data were analyzed using SPSS version 26.0 (IBM Corp., Armonk, NY, USA). All continuous variables underwent normality testing via Shapiro–Wilk test (α=0.05). Continuous data with normal and non-normal distributions were expressed as mean ± standard deviation (SD) and interquartile range, respectively, while categorical data are expressed as frequency. Within-group comparisons were performed using paired *t*-tests. Between-group differences in STAI-state change (ΔSTAI-state) were analyzed, and the mean difference with 95% confidence intervals (CI) was reported. Analysis of covariance (ANCOVA) was conducted with follow-up STAI-state as the dependent variable, group assignment as the independent variable, and baseline STAI-state as a covariate. Multivariable logistic regression analysis was performed to identify factors associated with moderate-to-severe anxiety (defined as STAI-state ≥40 at 3 months). Covariates included age, sex, educational level, STAI-trait score, VFQ-25 score, and comorbidities. A sensitivity analysis including group assignment as a predictor was also performed. A two-sided *p*-value < 0.05 was considered statistically significant.

## 3 Results

### 3.1 The characteristics of participants

None of the participants had a medical education background. A total of 169 and 170 participants were recruited into the intervention and control groups, respectively. In the intervention group, 12 participants (7.1%) were lost to follow-up, while 18 participants (10.6%) in the control group were lost to follow-up. Ultimately, 157 and 152 participants were included in the intervention and control groups, respectively. The demographic and clinical characteristics of the participants are presented in Table 1. There were no statistically significant differences between the groups in terms of sex, age, education level, duration of symptoms, shape of floaters, presence of photopsia, trait anxiety, or other clinical and demographic characteristics (*p* > 0.05), indicating good baseline comparability.

**Table 1:** The demographic and climnical characteristics of the participants.

Variables	Experimental group (*n* = 157)	Control group (*n* = 152)	*P*
15.6-7.4,-1.3498pt Age (Y)	53.47 ± 12.64	51.86 ± 12.73	0.266
Sex			
Male	84 (53.5%)	82 (53.9%)	0.938
15.6-7.4,-1.3498pt Female	73 (46.5%)	70 (46.1%)	
Education			
Primary or below	13 (8.3%)	19 (12.5%)	0.477
Middle/high school	75 (47.8%)	69 (45.4%)	
College or above	69 (43.9%)	64 (42.1%)	
15.6-7.4,-1.3498pt Duration of VF (M)	2.81 ± 3.99	2.58 ± 3.22	0.577
Flash			
No	129 (82.17%)	121 (79.61%)	0.567
Yes	28 (17.83%)	31 (20.39%)	
15.6-7.4,-1.3498pt Time of using electronic products daily (H)	2.89 ± 2.08	2.90 ± 2.04	0.968
Shape of floaters			
Punctiform	35 (22.3%)	40 (26.3%)	0.41
Filiform	122 (77.7%)	112 (73.7%)	
STAI-trait	26.60 ± 6.13	25.73 ± 5.37	0.187
15.6-7.4,-1.3498pt Comorbidities			
Hypertension			
No	146 (93%)	138 (90.8%)	0.477
15.6-7.4,-1.3498pt Yes	11 (7%)	14 (9.2%)	
Diabetes mellitus			
No	145 (92.4%)	143 (94.1%)	0.548
15.6-7.4,-1.3498pt Yes	12 (7.6%)	9 (5.9%)	
Myopia			
No	89 (56.7%)	84 (55.3%)	0.055
≤ -3.00 D	26 (16.6%)	21 (13.8%)	
−3.00 D~-6.00 D	23 (14.6%)	13 (8.6%)	
15.6-7.4,-1.3498pt >−6.00 D	19 (12.1%)	34 (22.4%)	
Presbyopia			
No	95 (60.5%)	90 (59.2%)	0.779
≤ +1.25 D	18 (11.5%)	19 (12.5%)	
+1.25 D~+2.00 D	44 (28%)	43 (28.3%)	

Y, years; VF, vitreous floaters; M, months; H, hours; D, diopters.

### 3.2 The effectiveness of multimedia health education on anxiety and VRQoL

**Figure 2:**
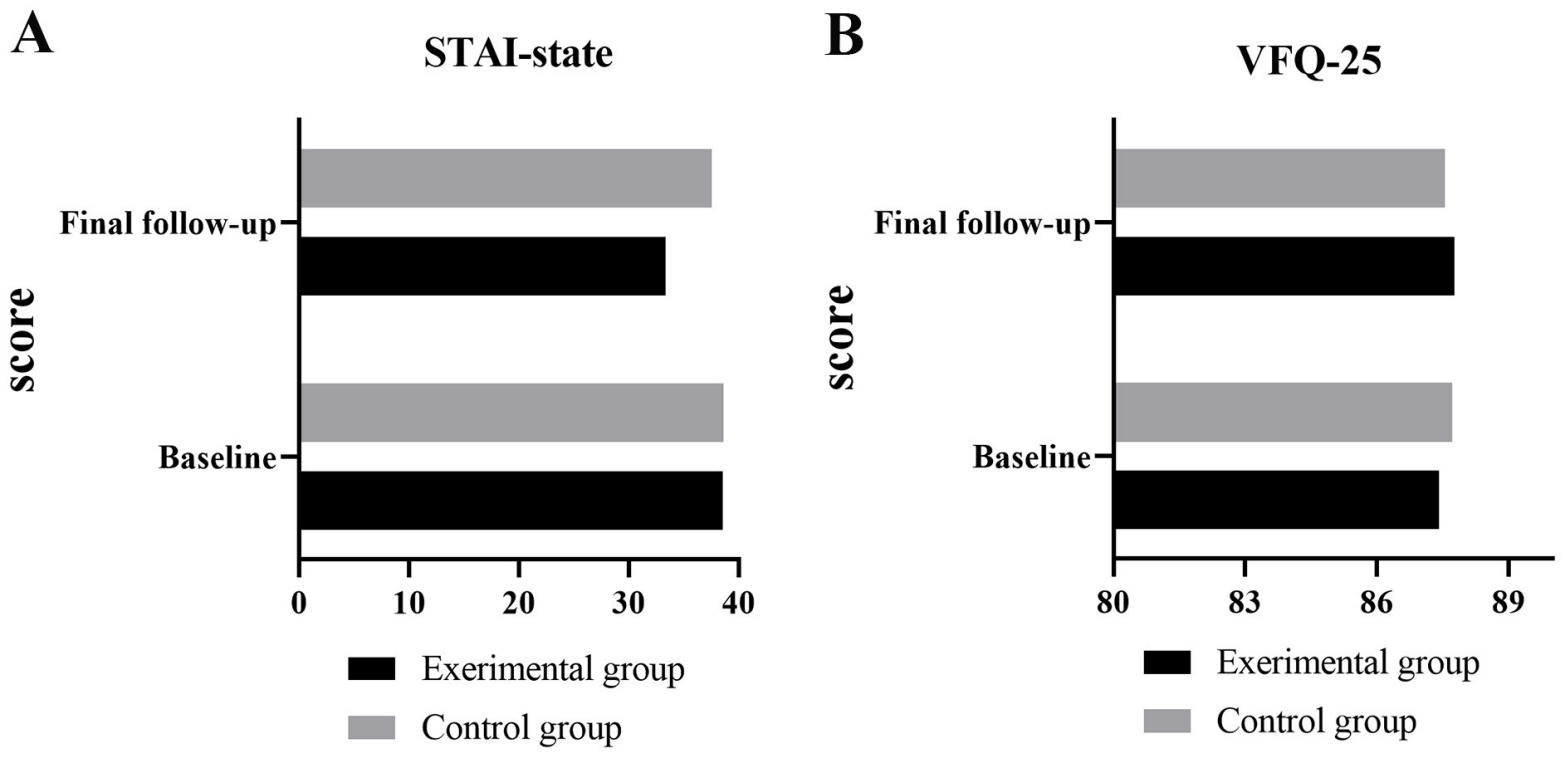
The effectiveness of intervention. **(A)**: STAI-state score; **(B)**: VFQ-25 score.

The follow-up questionnaire showed that the STAI-state scores of both the intervention and control groups decreased, with statistically significant differences (Experimental group: 38.57 ± 5.06 vs. 33.35 ± 6.47, *p* < 0.001; Control group: 38.64 ± 5.13 vs. 37.55 ± 6.21, *p* < 0.001, Figure 2A). However, there were no statistically significant differences in the changes in VFQ-25 scores between the two groups (Figure 2B). The improvement rate of STAI-state in the intervention group was significantly higher than that in the control group, with a statistically significant difference (13.22% ± 15.19% vs. 2.79% ± 10.18%, *p* < 0.001). Further analysis using ANCOVA, adjusting for baseline STAI-state, confirmed that the intervention group had significantly lower follow-up STAI-state scores compared with the control group (*p* < 0.001).

### 3.3 Stratified analysis by educational level

Within the intervention group, participants were stratified into three subgroups based on educational level (primary or below, secondary, and college or above). All subgroups demonstrated significant reductions in STAI-state scores after multimedia health education (all *p* < 0.05).

The absolute reduction in STAI-state scores was greater in participants with higher educational levels. Specifically, patients with a college degree or above showed a significantly greater reduction compared with those with primary education or below (5.39 ± 5.48 vs. 3.08 ± 6.47, *p* < 0.05).

### 3.4 Factors associated with anxiety severity

Based on STAI-state scores at 3-month follow-up, participants were categorized into mild anxiety and moderate-to-severe anxiety groups using a cutoff value of 40.

Univariate analysis showed significant differences between the two groups in age, educational level, presence of photopsia, VFQ-25 scores, STAI-trait, and diabetes status (Table 2). Patients with moderate-to-severe anxiety were older, had higher trait anxiety scores, and were more likely to have diabetes and photopsia, whereas those with mild anxiety had higher baseline VFQ-25 scores and greater improvement.

**Table 2:** The demographic and clinical characteristics of different levels of anxiety.

Variables	Mild *n* = 168	Moderate to severe *n* = 141	*P*
15.6-7.4,-1.3498pt Age	49.99 ± 12.70	55.89 ± 11.95	^***^
Sex			
Male	98 (58.3%)	68 (48.2%)	0.076
15.6-7.4,-1.3498pt Female	70 (41.7%)	73 (51.8%)	
Education			
Primary or below	26 (15.5%)	6 (4.3%)	0.004
Middle/high school	71 (42.3%)	73 (51.8%)	
College or above	71 (42.3%)	62 (44%)	
15.6-7.4,-1.3498pt Duration of VF (M)	2.5813 ± 3.40	2.844 ± 3.90	0.528
Flash			
No	147 (87.5%)	103 (73%)	0.001
15.6-7.4,-1.3498pt Yes	21 (12.5%)	38 (27%)	
Shape of floaters			
Punctiform	48 (28.6%)	27 (19.1%)	0.054
Filiform	120 (71.4%)	114 (80.9%)	
VFQ-25	88.863 ± 4.78	86.004 ± 5.24	^***^
STAI-trait	23.61 ± 5.085	29.22 ± 5.036	^***^
ΔSTAI-state	3.0357 ± 5.29	3.3617 ± 5.13	0.585
15.6-7.4,-1.3498pt ΔVFQ-25	0.9286 ± 5.95	−1.322 ± 5.92	0.001
Hypertension			
Yes	11 (6.5%)	14 (9.9%)	0.278
15.6-7.4,-1.3498pt No	157 (93.5%)	127 (90.1%)	
Diabetes mellitus			
Yes	6 (3.6%)	15 (10.6%)	0.014
15.6-7.4,-1.3498pt No	162 (96.4)	126 (89.4%)	
Myopia			
No	84 (50%)	89 (63.1%)	0.117
≤ -3.00 D	27 (16.1%)	20 (14.2%)	
−3.00 D~-6.00 D	23 (13.7%)	13 (9.2%)	
15.6-7.4,-1.3498pt >−6.00 D	34 (20.2%)	19 (13.5%)	
Presbyopia			
No	112 (66.7%)	73 (51.8%)	0.273
≤ +1.25 D	17 (10.1%)	20 (14.2%)	
+1.25 D~+2.00 D	39 (23.2%)	48 (34%)	

M, monthes; D, Diopter; ^***^, *P* < 0.001; Δ, defined as the difference between pre-intervention and post-intervention scores (pre-intervention minus post-intervention).

Multivariable logistic regression analysis (Table 3) identified several independent factors associated with anxiety severity at follow-up. Lower educational level was associated with a significantly lower likelihood of moderate-to-severe anxiety compared with college or above (primary or below: OR = 0.09, 95% CI: 0.025–0.323, *p* < 0.001). Increasing age was associated with higher odds of moderate-to-severe anxiety (OR = 1.05 per year, 95% CI: 1.02–1.08, *p* = 0.001). Higher STAI-trait scores were also strongly associated with increased anxiety severity (OR = 1.27, 95% CI: 1.19–1.36, *p* < 0.001). In addition, patients without diabetes had significantly lower odds of moderate-to-severe anxiety compared with those with diabetes (OR = 0.226, 95% CI: 0.056–0.911, *p* = 0.036).

**Table 3:** The model after adjusting for significant variables.

Variables	β	*P*	OR (95% CI)
Education		0.001	
Primary or below	−2.406	^***^	0.09 (0.025, 0.323)
Middle/high school	−0.356	0.276	0.701 (0.37, 1.328)
College or above	0		
Age	0.049	0.001	1.05 (1.021, 1.08)
VFQ-25	−0.089	0.004	0.915 (0.861, 0.972)
15.6-7.4,-1.3498pt STAI-trait	0.241	^***^	1.272 (1.191, 1.359)
Diabetes mellitus			
No	−1.486	0.036	0.226 (0.056, 0.909)
15.6-7.4,-1.3498pt Yes	0		
Flash			
No	−0.505	0.18	0.604 (0.289, 1.263)
Yes	0		

^***^, *P* < 0.001.

In a sensitivity analysis including group assignment as a covariate, the intervention group remained significantly associated with a lower likelihood of moderate-to-severe anxiety, confirming the robustness of the findings.

## 4 Discussion

The psychological burden associated with VF represents an emerging research frontier, with preliminary data suggesting a bidirectional relationship. VF exerts detrimental psychological effects manifesting as elevated depression and anxiety levels (8). Wagle et al. quantitatively assessed the impact of health-related quality of life (HRQoL) on VF in a cohort of 266 patients using standardized utility value metrics, and demonstrated that symptomatic degenerative VF significantly impairs HRQoL. Notably, the cohort exhibited a mean willingness-to-risk threshold equivalent to accepting a 11% mortality risk and 7% blindness risk for complete symptom resolution, a risk tolerance comparable to that observed in the diabetic retinopathy and age-related macular degeneration cohorts (25). While the HRQoL burden of VF appears less severe than that of advanced ocular or systemic diseases, its population-level impact remains important, given its high prevalence. These findings emphasize the necessity for stratified management based on symptom severity-indexed visual disturbances.

The current management of VF includes PPV, Nd:YAG laser vitreolysis, and follow-up to monitor disease progression (26). Although PPV is effective in alleviating both floaters and associated psychological distress, its invasive nature carries substantial complication risks (27, 28). Crucially, the lack of validated objective parameters for surgical candidacy necessitates rigorous preoperative risk-benefit stratification. Nd:YAG laser vitreolysis shows promise in select cases, with cohort studies reporting significant VRQoL enhancement and anxiety reduction at the 10-day follow-up (2). However, current evidence for the clinical use of Nd:YAG in the treatment of VF is limited and paradoxical. The conflicting results and potentially vision-threatening complications of published studies have sparked controversy, possibly preventing widespread adoption of this technology from being widely adopted. For patients without functional visual impairment, observation remains first-line approach (3). However, because of its natural degeneration and kindness, many clinicians may overlook the importance of paying attention to patients' mental health status.

Some patients experiencing flashes and spots in perception frequently consult ophthalmologists, but there are no answers, primarily because these symptoms are not necessarily related to ocular pathology. Cipolletta et al. believed that individuals' experiences and reactions to floaters may vary depending on their perceptions of their condition, trust in medicine, and psychological resilience (10). A psychological perspective may provide a viable analytical framework for patients with VF who lack definitive pathological correlates. In this study, anxiety levels significantly improved following multimedia health education among patients with VF, whereas the VFQ-25 scores demonstrated non-significant improvements. Further large-scale longitudinal studies are necessary to validate the relationship between psychological status and VRQoL. Studies have demonstrated the effectiveness of health education in alleviating psychological anxiety among patients with certain conditions. Kuo et al. found that multimedia health education interventions effectively reduced anxiety in patients undergoing mammography (14). Similarly, during the COVID-19 pandemic, Zemni et al. conducted a randomized controlled trial that confirmed the value of health education in alleviating anxiety in patients under COVID-19 quarantine (12). To the best of our knowledge, there are currently no studies on the use of health education to alleviate anxiety in patients with VF.

In the present study, we evaluated the effect of a structured multimedia health education program on anxiety in patients with VF. The results demonstrated that both standard post-examination counseling and multimedia education were associated with reductions in STAI-state scores; however, the magnitude of improvement was significantly greater in the multimedia group. Between-group analysis further confirmed that the reduction in anxiety was significantly larger in the intervention group, and this effect remained robust after adjustment for baseline anxiety levels. These findings suggest that not only reassurance, but also the format, depth, and clarity of information delivery, play a critical role in alleviating anxiety.

From a psychological perspective, anxiety in patients with VF appears to be driven largely by uncertainty and misinterpretation of symptoms. Many patients perceive floaters as indicative of serious ocular pathology, such as retinal detachment or diabetic retinopathy. Although routine verbal counseling provides reassurance, it is often brief and lacks structured explanation. In contrast, multimedia education integrates visual, textual, and auditory information to present complex medical knowledge in a more accessible and comprehensible manner. This structured approach may enhance patients' understanding of disease mechanisms, improve their sense of control, and reduce uncertainty, thereby alleviating anxiety (29).

Interestingly, our findings revealed that lower educational level was associated with a reduced likelihood of moderate-to-severe anxiety, whereas patients with higher educational attainment exhibited greater anxiety reduction following multimedia education. Although this may appear counterintuitive, several mechanisms may explain this phenomenon. Individuals with higher education may have greater access to health-related information, including internet- and AI-based sources, and may be more prone to overinterpret or catastrophize benign symptoms. In addition, higher visual demands in professional or academic settings may increase symptom awareness and psychological burden. Conversely, individuals with lower educational levels may be less exposed to complex or potentially misleading information, resulting in lower baseline anxiety. Importantly, once provided with structured and comprehensible medical explanations, patients with higher education may benefit more from multimedia interventions due to better cognitive assimilation of the information.

Consistent with previous studies, we also found that older age, higher trait anxiety, and the presence of diabetes were independently associated with increased anxiety severity. In particular, patients with diabetes may misattribute vitreous floaters to diabetic retinopathy due to prior health education emphasizing retinal complications, thereby increasing psychological distress. These findings highlight the importance of individualized communication strategies that take into account patients' demographic and psychological characteristics.

Another important implication of this study is that physician–patient communication itself represents a form of clinical intervention. Both groups in our study received post-examination counseling; however, the multimedia group received a more structured and comprehensive form of communication. This suggests that optimizing the quality and delivery of information may be as important as clinical decision-making in managing conditions where reassurance is the primary treatment approach.

Several limitations should be acknowledged. First, this study employed a quasi-randomized temporal allocation based on calendar week rather than strict individual-level randomization, which may introduce potential time-related confounding, such as seasonal variation or differences in clinic flow. However, consistent eligibility criteria, standardized examination procedures, and stable clinical personnel were maintained throughout the study period to minimize bias. Second, baseline anxiety was assessed after completion of the ophthalmic examination, which may have already alleviated some degree of pre-existing anxiety and therefore may underestimate the true baseline anxiety level.

Third, the multimedia intervention was delivered by a senior vitreoretinal specialist, and the observed effect may partially reflect an authority or trust effect associated with experienced clinicians. Future studies are warranted to evaluate whether similar outcomes can be achieved when the intervention is delivered by general ophthalmologists or trained mid-level providers, which would enhance the generalizability and scalability of this approach. Finally, several potential confounding factors were not systematically assessed, including additional healthcare-seeking behavior during follow-up, use of medications affecting anxiety, and differences in access to external health information sources such as the internet or AI-based platforms. These factors may have influenced anxiety outcomes and should be addressed in future studies.

In conclusion, unnecessary anxiety in patients with VF appears to arise primarily from insufficient understanding of the condition and misinterpretation of symptoms. Our findings demonstrate that structured multimedia health education is more effective than routine verbal counseling in alleviating anxiety. This approach provides an accessible and non-invasive strategy to improve psychological wellbeing and highlights the importance of integrating standardized patient education into routine clinical practice.

## Data Availability

The original contributions presented in the study are included in the article/Supplementary material, further inquiries can be directed to the corresponding author.
